# *De novo* COVID-19-associated insulin resistance drives dysregulated neutrophil extracellular trap formation (NETosis) four months after infection

**DOI:** 10.3389/fimmu.2026.1787799

**Published:** 2026-05-04

**Authors:** Sergio Sanhueza, Camilo Cabrera, Romina Quiroga, Bárbara Antilef, Camila Muñoz, Agustín Vera, Ricardo Cartes, Liliana Lamperti, Enrique Guzmán-Gutiérrez, Claudio Aguayo, Valeska Ormazábal, Mauricio Alejandro Hernández, Jaime Lastra, Benilde Riffo, Gustavo Cerda, Luciano Ferrada, David De Gonzalo-Calvo, María C. García-Hidalgo, Mario Henríquez, María Inés Barría, Ricardo A. Verdugo, Alicia Colombo, Gonzalo Labarca, Estefanía Nova-Lamperti

**Affiliations:** 1 Molecular and Translational Immunology laboratory, Department of Clinical Biochemistry and Immunology, Pharmacy Faculty, University of Concepcion, Concepcion, Chile; 2 Facultad de Odontología, Universidad San Sebastián, Concepción, Chile; 3 Facultad de Derecho y ciencias sociales, Universidad San Sebastián, Concepción, Chile; 4 Division of Biotechnology, MELISA Institute, San Pedro de la Paz, Chile; 5 Medicine Department, Hospital Guillermo Grant Benavente and Medicine Faculty, University of Concepcion, Concepcion, Chile; 6 Prevegen Laboratory, Concepcion, Chile; 7 Advanced Microscopy Center, University of Concepcion, Concepcion, Chile; 8 Clinical and Molecular Phenotyping, Biomedical Research Institute of Lleida – Dr. Pifarré Foundation, IRBLleida, Lleida, Spain; 9 Centro de Investigación Biomédica en Red of Respiratory Diseases (CIBERES), Institute of Health Carlos III, Madrid, Spain; 10 Magíster en Ciencias de la Motricidad Humana, Universidad Adventista de Chile, Chillán, Chile; 11 Escuela de Kinesiología, Facultad de Salud, Universidad Santo Tomás, Los Ángeles, Chile; 12 Departamento de Enfermedades Respiratorias, Facultad de Medicina, Pontificia Universidad Católica de Chile, Santiago, Chile; 13 Facultad de Medicina, Universidad San Sebastián, Puerto Montt, Chile; 14 Departamento de Oncología Básico Clínico and Centro para la Prevención y el Control del Cáncer (CeCAN), Facultad de Medicina, Universidad de Chile, Santiago, Chile; 15 Biobanco de tejidos y fluidos, Universidad de Chile, Santiago, Chile; 16 Division of Sleep Medicine, Brigham and Women’s Hospital, Harvard Medical School, Boston, MA, United States; 17 Internal Medicine, Complejo Asistencial Dr. Víctor Ríos Ruiz, Los Ángeles, Chile

**Keywords:** COVID-19, insulin resistance, long-covid, NETosis, neutrophil

## Abstract

**Background:**

Glucose metabolism disorders (GMDs) are established risk factors for severe COVID-19, but increasing evidence indicates that they may also develop *de novo* after SARS-CoV-2 infection. Neutrophil extracellular trap formation (NETosis) plays a central role in immunothrombosis, and because neutrophils rely predominantly on glycolysis, they are particularly sensitive to systemic metabolic disturbances. However, the impact of post-COVID-19 GMDs on NETosis remains poorly understood. This study aimed to characterize the emergence of GMDs after COVID-19 and to determine their effect on neutrophil NETosis.

**Methods:**

Sixty COVID-19 patients were stratified according to the presence or absence of GMDs before infection and at four months post-infection. Demographic, clinical, metabolic, and inflammatory parameters were assessed. Vital NETosis was quantified by flow cytometry. In addition, the capacity of patient plasma to induce NETosis was evaluated using live-cell imaging of healthy neutrophils as biosensors.

**Results:**

Among patients without pre-existing GMDs, 24 of 36 developed insulin resistance (IR) four months after COVID-19. Neutrophils from these patients exhibited increased basal NETosis but showed impaired NETosis in response to TLR7/8 agonists, key sensors of viral single-stranded RNA, compared with control groups. In contrast, NETosis responses to IL-6 and TNF-α were preserved, excluding an intrinsic neutrophil defect. Plasma from IR patients significantly enhanced NETosis, and *in vitro* experiments demonstrated that insulin enhances NETosis independently of glucose concentrations.

**Discussion:**

*De novo* IR following COVID-19 dysregulates NETosis primarily through an insulin-enhancing effect. Post-viral control of glucose metabolism disorders may be critical to limit pathological NETosis and its thrombo-inflammatory consequences.

## Graphical Abstract

**Figure:**
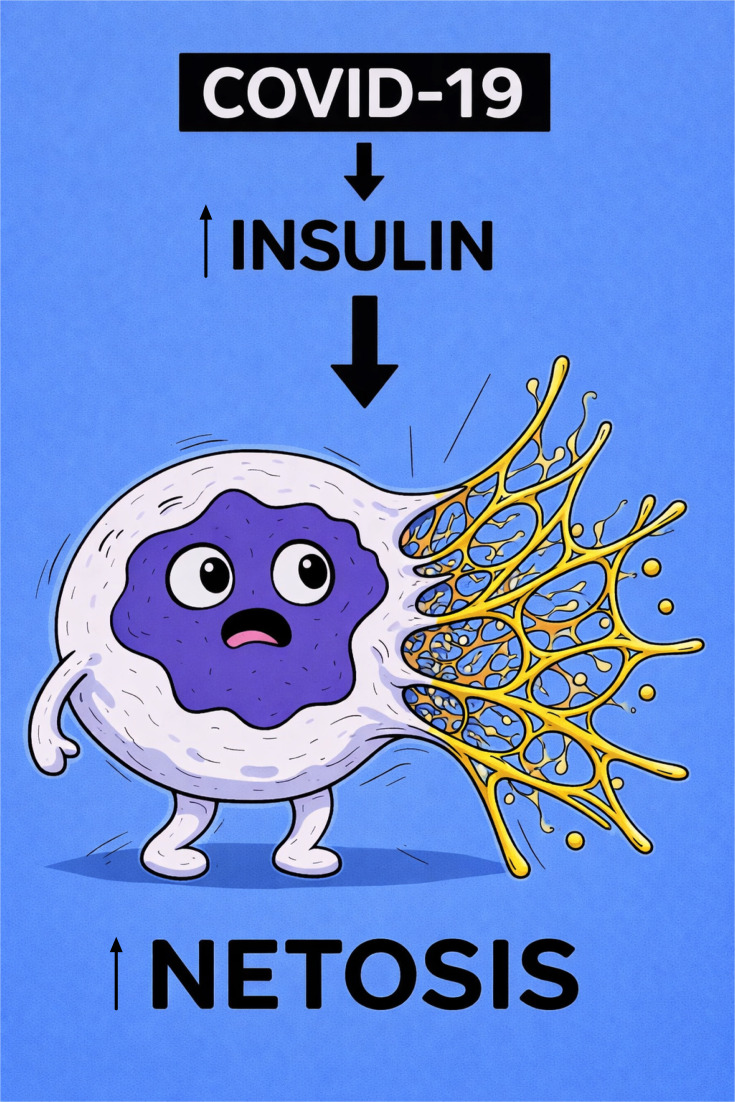

## 1 Introduction

COVID-19, caused by infection with SARS-CoV-2, ranges from mild illness to severe disease characterized by pneumonia, hypoxemia and acute respiratory distress syndrome (ARDS) (1). Glucose metabolism disorders (GMDs) are established comorbidities associated with poorer COVID-19 outcomes. For example, prediabetes has been associated with increased COVID-19 mortality (HR = 3.3) (2), and a higher risk of severe disease (OR = 2.58) (3). In Wuhan, another study found a significant association between the triglyceride-glucose index (TyG) and increased severity and mortality risk (OR = 2.9) (4). Beyond the impact of pre-existing GMDs, COVID-19 has been associated with the development of insulin resistance (IR) in previously normoglycemic individuals. A study from Wenzhou reported increased IR markers, including C-peptide, Homeostatic Model Assessment for Insulin Resistance (HOMA-IR), and the TyG index following infection (5), and longitudinal data further indicate that normoglycemic patients may develop hyperglycemia and IR up to six months after COVID-19 (6). Severe COVID-19 is also characterized by neutrophilia, supporting a pivotal role for neutrophils in disease pathogenesis, including the contribution of neutrophil extracellular traps (NETs) (7–11). Elevated circulating NETs levels are associated with severe COVID-19, and serum from these COVID-19 patients induces increased NET formation in healthy neutrophils compared with control serum (12). Neutrophil extracellular trap formation (NETosis) is a key driver of immunothrombosis and is tightly coupled to cellular glycolytic metabolism, rendering neutrophils highly sensitive to systemic metabolic alterations. However, how GMDs emerging after COVID-19 affect NETosis remains unclear. This study investigated the development of post-COVID-19 GMDs and their impact on NETosis.

## 2 Methods

### 2.1 Study design

An observational and prospective cohort study was conducted following the recommendations of the STROBE declaration (13). The study was approved by the Institutional Ethics Committee of the Biobio (IRB: CEC113) and Concepción (IRB: CEC-SSC:20-07-26) Health Services, and Universidad de Concepción (CEBB 676-2020). Patients with COVID-19 were recruited from the Víctor Ríos Ruiz Hospital, between April and July 2020. The diagnosis of COVID-19 was confirmed by PCR or radiological image positive for SARS-CoV-2. Four months after the acute phase, patients were recruited again. COVID-19 patients were between 18 and 70 years old. All participants were not vaccinated during the acute phase or at 4 months post-COVID-19; however, they were vaccinated between 9- and 12-months post-infection. Patients over 70 years of age and patients who did not attend post-acute phase recruitment, were transferred to another hospital or city after discharge, were in palliative care, required persistent oxygen or mechanical ventilation, had decompensated chronic comorbidities, or had a mental disability that prevented completion of assessments were not included in the study. Finally, pregnant women were also excluded during the acute phase or during follow-up. All participants signed informed consent before entering the study, and all methods were performed in accordance with the Declaration of Helsinki and Good Clinical Practice. Graphical images were created with BioRender.com (https://BioRender.com/e04ucpw).

### 2.2 Clinical data

**Figure 1:**
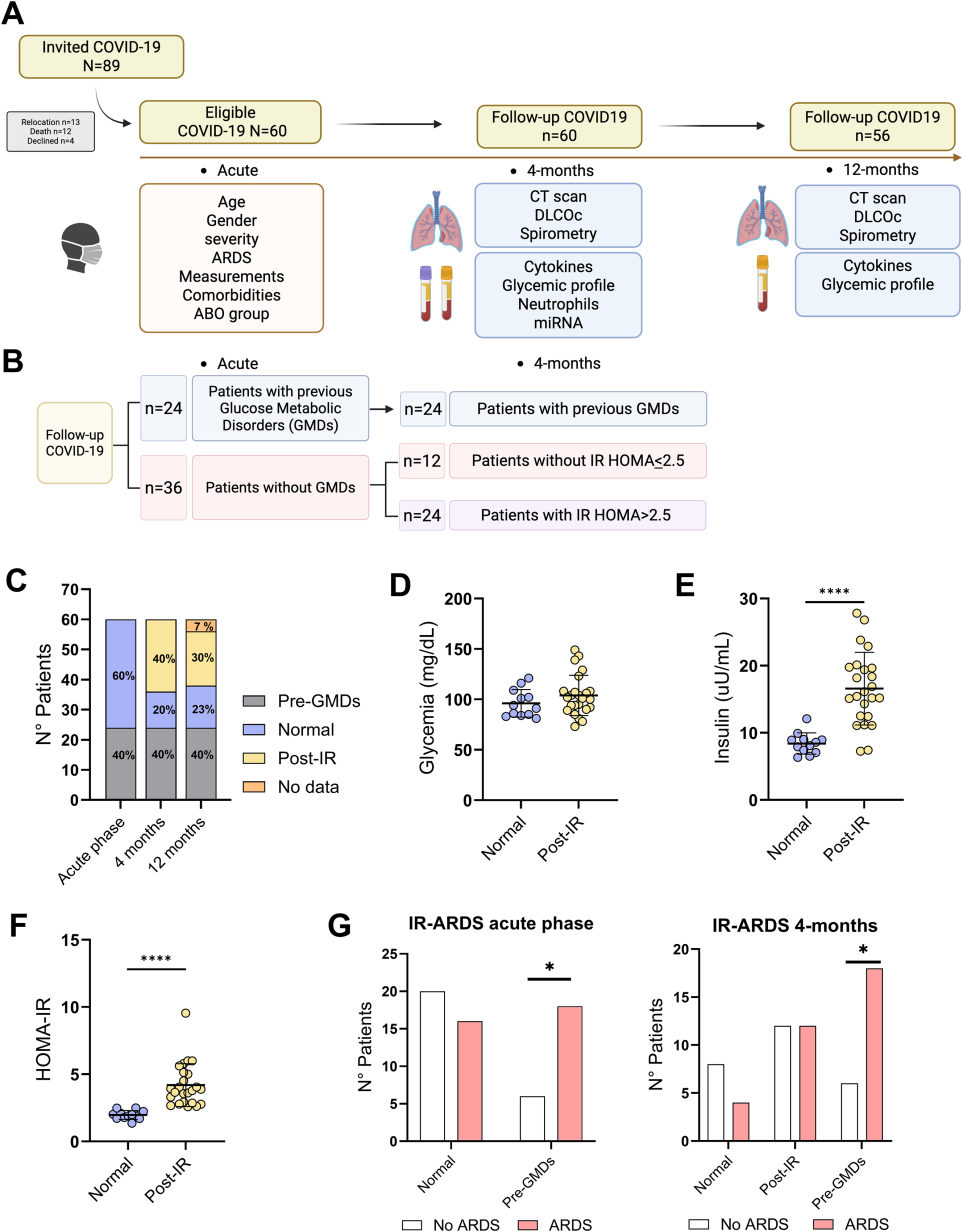
Study design flowchart. **(A)** A total of 89 patients with a confirmed diagnosis of COVID-19 were invited to participate in the study, of whom 29 were excluded, resulting in a study cohort of 60 patients. Clinical and demographic data were collected during the acute phase and 4- and 12-months post-COVID-19. **(B)** Distribution flow of patients from the acute phase to 4 months post-COVID-19 according to the prior presence of GMDs or the development of post-COVID-19 IR. IR is determined by calculating HOMA-IR with values greater than 2.5. **(C)** Bar chart of the distribution of the cohort of 60 patients during the acute phase of COVID-19, 4 months post-COVID-19, and 12 months post-COVID-19, using the presence of GMDs before COVID-19 or the development of post-COVID-19 IR as the criterion. **(D)** Scatter plots comparing patients without GMDs versus patients who developed IR 4 months post-COVID-19 based on glycemia, **(E)** Insulinemia and **(F)** HOMA-IR. **(G)** Bar chart representing the distribution of patients during the acute stage and 4 months post-COVID-19 according to the groups in our cohort categorized as normal glucose metabolism, pre-GMDs and post-IR based on the development of ARDS during COVID-19. For panels **(D–F)** Unpaired t-test ****p <0.0001. For panel **(G)** Chi-square *p <0.05.

After being invited to participate in the study, sixty patients with COVID-19 provided written informed consent. They experienced the disease with different degrees of severity between March and June 2020 (**Figure 1A**). Patients were recruited at 4 and 12 months post-acute phase, evaluating them clinically, reporting age, gender, ABO blood group, measurements, body mass index, smoking history, alcohol consumption, disease severity according to World Health Organization (WHO) guidelines, development of ARDS during the acute phase of the pathology, symptoms, previous comorbidities. Also, four months following their COVID-19 diagnosis, peripheral blood samples were collected. Biochemical and lipid profiles, complement proteins, and complete blood count examinations were performed. Plasma derived from these patient samples was preserved at -80 °C for further experiments. RNA isolation and plasma microRNA (miRNA) quantification were performed as previously described (14, 15). Patients were classified according to the presence of IR, dividing them into 3 groups consisting of participants without GMDs, patients with GMDs prior to COVID-19, and patients who developed post-COVID-19 IR (4 months) (**Supplementary Table 1**). Altered parameters were considered as: elevated glucose levels as value greater than 100 mg/dL (5.55 mmol/L), insulin levels above 16 µU/mL, and a HOMA-IR index exceeding 2.5, indicating insulin resistance. Any patient not exhibiting these specific conditions was classified as having no GMDs. Additionally, the evolution of glucose profile alterations was evaluated 12 months following infection.

### 2.3 NETosis analysis in isolated polymorphonuclear cell

PMNs isolation was performed from 5 mL peripheral blood obtained through venous puncture from 60 COVID-19 patients, following the protocol suggested by the manufacturer (Axis-Shield), 702 g for 30 minutes at room temperature in a swing-out rotor centrifuge (Changsha Xiangyi centrifuge L-550). After centrifugation, PMN were washed at 400 g for 15 minutes and resuspended in warm X-vivo medium with 2pM Ca2+. After isolation, 1x10^5^ PMN cells were used to determine the purity of the sample by flow cytometry with SIGLEC8 (Catalog No.347106, Biolegend) and CD16 (Catalog No.302016, Biolegend) staining. 1x10^5^ PMN cells were used to determine TLR7 and TLR8 expression by flow cytometry with anti-TLR7 (Catalog No.MA5-16249, Invitrogen) and anti-TLR8 (Catalog No.MA5-16194, Invitrogen) staining with intracellular staining (Catalog No.552300, Invitrogen). For NETosis, 1 x 10^6^ cells were incubated for 30 minutes at 37 °C and 5% CO2 under basal conditions or using IL-6 (20ng/mL) and TNFα (2ng/mL), or TLR7/8 agonists (CL075 (1μg/mL), ImiQ (2μg/mL), and R848 (2μg/mL), ssRNA40 (1μg/mL) and ssRNA 41(1μg/mL) (Catalog No.tlrl-kit3hw3, InvivoGen). Cells were labeled with LIVE/DEAD™ Far Red for 10 min in the dark (Catalog No.L10120, Invitrogen). 15 minutes before the flow cytometry reading, 30 nM Sytox Blue (Catalog No.S11348, Invitrogen) was added at room temperature in the dark. Samples were acquired on the LSR Fortessa X20 flow cytometer (BD Biosciences, USA), and the data were analyzed using FlowJo software (Tree Star Inc.).

### 2.4 Quantification of NETosis in healthy neutrophils exposed to plasma from COVID-19 patients by live-cell imaging

A sample of 50 μL containing 1 x 10^4^ PMNs in X-vivo medium supplemented with 2 pM CaCl2 was added to each well of a 96-well plate, which was clear and flat-bottomed. Subsequently, 50µL of different plasmas from control or COVID-19 patients in their acute phase and post-infection sequelae were added. Finally, the Sytox Green (Thermofisher) was added at 30 nM, and analyzed by IncuCyte S3 (Essen Bioscience, Sartorius) for real-time microscopic monitoring. The percentage of NETosis was automatically calculated using the IncuCyte cell-by-cell analysis module by dividing the number of Sytox+ cells by the total number of cells and expressing the result as a percentage.

### 2.5 Evaluation of the glucose effect on NETosis production in healthy human neutrophils in the presence and absence of insulin

The previously described flow cytometry protocol using Live/Dead dye and Sytox Blue was applied to quantify NETosis. DMEM medium without glucose supplementation was used, and 8 equal populations of 1 x 10^6^ cells were exposed to different glucose concentrations: 4 mmol/L (72 mg/dL), 5.5 mmol/L (100 mg/dL), 7.5 mmol/L (135 mg/dL), and 25 mmol/L (450 mg/dL). These 8 groups of neutrophils were divided into those exposed to insulin at 10 µU/mL and those not exposed to insulin, with the incubation carried out for 1 hour at 37 °C and 5% CO2. For vital NETosis, images of Sytox Green in neutrophils at 4 mmol/L (72 mg/dL) without and with insulin (10 µU/mL) were analyzed using confocal microscopy. High-resolution images were obtained using live-cell confocal microscopy, as previously described by us (16, 17). Briefly, neutrophils were seeded onto Cellview cell culture slides with a glass bottom, Advanced TC (Catalog No.543979, Greiner Bio-One). After treatments, cells were pre-stained with Hoechst 33342 (0.1 μg/mL) as a nuclear dye and CellMask Deep Red (0.3×) as a plasma membrane marker (both from Molecular Probes) for 30 min. Cells were then washed and incubated with Sytox Green (30nM) diluted in complete medium as a NETosis marker and imaged using a Leica SP8 confocal microscope equipped with Lightning super-resolution, CO_2_ and temperature control modules, and three ultrasensitive hybrid detectors (HyD). Images were acquired in x, y, and z at 1024 × 1024 pixels and are shown as 3D reconstructions generated using LAS X software.

### 2.6 Data analysis

Statistical analysis of the data was performed using the statistical analysis software GraphPad Prism. Normality was determined using Anderson-Darling, D’Angostino & Pearson, Shapiro-Wilk, Kolmogorov-Smirnov tests. Subsequently, differences between groups were assessed using parametric or non-parametric analysis of variance (ANOVA or Kruskall-Wallis, respectively) as appropriate, including Welch’s correction for unequal standard deviations among data, with a p < 0.05 considered statistically significant for the statistical comparisons by ANOVA. For multiple comparisons, Dunnet T3 multiple test correction was applied. For comparisons of categorical variables, the Chi-square test was used. Pearson or Spearman correlation analysis, as appropriate, was established for the data between parameters of carbohydrate metabolism, cytokines, and chemokines in the plasma of COVID-19 patients in their acute phase, post-infection sequelae, and the NETosis quantified by IncuCyte, considering a statistically significant correlation with a p-value < 0.05.

## 3 Results

### 3.1 Characterization of GDMs at 4 months post-infection in a cohort of COVID-19 patients

The characterization of glucose metabolism parameters was carried out in a cohort of 60 patients who survived COVID-19 (**Figure 1A**). During the acute phase of the disease, clinical and demographic data were collected, including sex, blood type, anthropometric measurements, medication use, treatment received, symptoms, and presence of pre-existing comorbidities (**Supplementary Table 1**). During the acute phase, 36 out of 60 patients had no reported GMDs, whereas 24 patients presented GMDs, including either type 2 diabetes mellitus or IR. All patients experienced mild, moderate, or severe COVID-19 according to WHO criteria (18, 19). Four months after infection, patients were re-evaluated through the assessment of clinical biochemical and inflammatory parameters. In addition, pulmonary function was evaluated by measuring the diffusing capacity of the lungs for carbon monoxide adjusted for hemoglobin (DLCOc) and by computed tomography (CT) scan (**Supplementary Table 1**). Based on the clinical biochemical evaluation, alterations in glucose metabolism profiles were identified (**Figure 1B**). Among the 36 patients initially without metabolic alterations, 24 developed *de novo* IR, whereas 12 maintained normal glucose metabolism (**Figure 1C**). Finally, at 12 months post-COVID-19, follow-up evaluation revealed dynamic changes in both patient groups: a subset of individuals with IR at 4 months showed metabolic improvement, whereas some patients without IR at this time point exhibited a deterioration in glucose metabolism parameters (**Supplementary Table 2**).

Subsequently, to identify specific characteristics associated with the cohort’s glycemic profile, statistical comparisons were made using several factors comprising the biochemical profile of the study group. First, glycemia, insulin, and HOMA-IR parameters identified four months after COVID-19 were evaluated. It was determined that the group of patients who developed IR after viral infection did not show an increase in blood glucose levels compared to the group of patients without GMDs (**Figure 1D**), unlike insulin levels (**Figure 1E**) and HOMA-IR (**Figure 1F**), where a significant increase was observed. HOMA-IR confirms the development of IR post-COVID-19. These data suggest that patients who develop IR post-COVID-19 can maintain stable glucose levels by increasing insulin secretion. Based on these differences, criteria related to the metabolic functioning of the cohort were evaluated to accurately identify the profile of the study group using circulatory parameters (**Table 1**). The analysis began with a panel of serum cytokines, identifying a significant increase in IL-6, IL-8, CXCL-9, and CXCL-10 in the group of patients who developed IR compared to the group without GMDs (**Table 1**). Similarly, it was also possible to evaluate proteins related to damage in organs that play a central role in human metabolism, such as the liver, kidneys, and pancreas, which are directly related to the development of IR (20, 21) Thus, a significant increase in aspartate aminotransferase (AST), alanine aminotransferase (ALT) and alkaline phosphatase (AP) was determined in the group of patients who developed IR compared to the group without GMDs (**Table 1**). Circulating miR-495-3p, previously associated with diabetes-related complications (22), was upregulated in the IR group when compared with the control group (**Table 1**). Other blood parameters such as uric acid, calcium, phosphorus, bilirubin, total protein, albumin, globulin, complete blood count, and lipid profile exhibited no significant differences between groups (**Supplementary Table 3**). Finally, to assess whether GMDs were related to ARDS, we analyzed the number of patients who developed ARDS between groups (**Figure 1G**). During the acute phase of the disease, patients with pre-existing GMDs were predisposed to ARDS, however, the development of IR post-COVID-19 was not related to the development of ARDS during the acute phase of COVID-19 (**Figure 1G**).

**Table 1:** Circulatory and inflammatory parameters and microR.

Circulating parameters	Patients without GMDs	Patients with insulin resistance post COVID-19	*p*-Value
Circulating parameters			
Cytokines (pg/mL)			
IL-12 (SD)	0.5942 (± 0.1066)	0.5833 (± 0.289)	n.s
TNF (SD)	0.4983 (± 0.4250)	0.3917 (± 0.4004)	n.s
IL-1β (SD)	0.2667 (± 0.1580)	0.2800 (± 0.1992)	n.s
IL-6 (SD)	1.054 (± 0.4238)	1.962 (± 1.001)	**
IL-8 (SD)	3.086 (± 1.898)	5.421 (± 2.857)	*
Chemokines (pg/mL)			
CXCL-10 (SD)	61.86 (± 21.12)	93.92 (± 40.83)	*
CXCL-9 (SD)	6.382 (± 3.142)	13.80 (± 11.27)	*
CCL-5 (SD)	380.8 (± 228.8)	464.3 (± 324.1)	n.s
CCL-2 (SD)	32.06 (± 18.25)	45.32 (± 33.39)	n.s
Damage proteins (U/L)			
CK (SD)	111.2 (± 54.92)	204.7 (± 93.49)	n.s
AST (SD)	16.92 (± 8.118)	25.75 (± 14.94)	**
ALT (SD)	17.75 (± 8.508)	33.38 (± 26.30)	*
GGT (SD)	19.36 (± 13.87)	32.25 (± 27.21)	n.s
AP (SD)	56.75 (± 24.44)	72.29 (± 19.49)	*
LDH (SD)	153.5 (± 50.73)	175.3 (± 49.47)	n.s
miR (log10_2_(ΔCq))	P value Normal vs Post IR	P value Normal vs Pre GMDs	P value Post IR vs Pre GMDs
miR9-5p	0.0220	0.0104	0.8448
miR-17-5p	0.0424	0.0772	0.5854
miR21-5p	0.0096	0.0253	0.5479
miR24-3p	0.0113	0.1401	0.1908
miR-27a-3p	0.1061	0.0284	0.5674
miR-126-3p	0.0259	0.0882	0.2969
miR-146a-5p	0.0138	0.0420	0.3435
miR221	0.1345	0.0128	0.7391
miR495-3p	0.0103	0.3846	0.0292

a) CK, creatine kinase; b) AST, aspartate aminotransferase; c) ALT, alanine aminotransferase; d) GGT, Gamma-glutamyl transferase; e) AP, alkaline phosphatase; f) LDH, lactate dehydrogenase; g) *n*, number of patients; h) SD, standard deviation; i) miR, Micro RNAs. Mann Whitney test; **p < 0.01 and *p < 0.05, n.s is not significant.

### 3.2 Post-COVID-19 IR dysregulate NETosis

**Figure 2:**
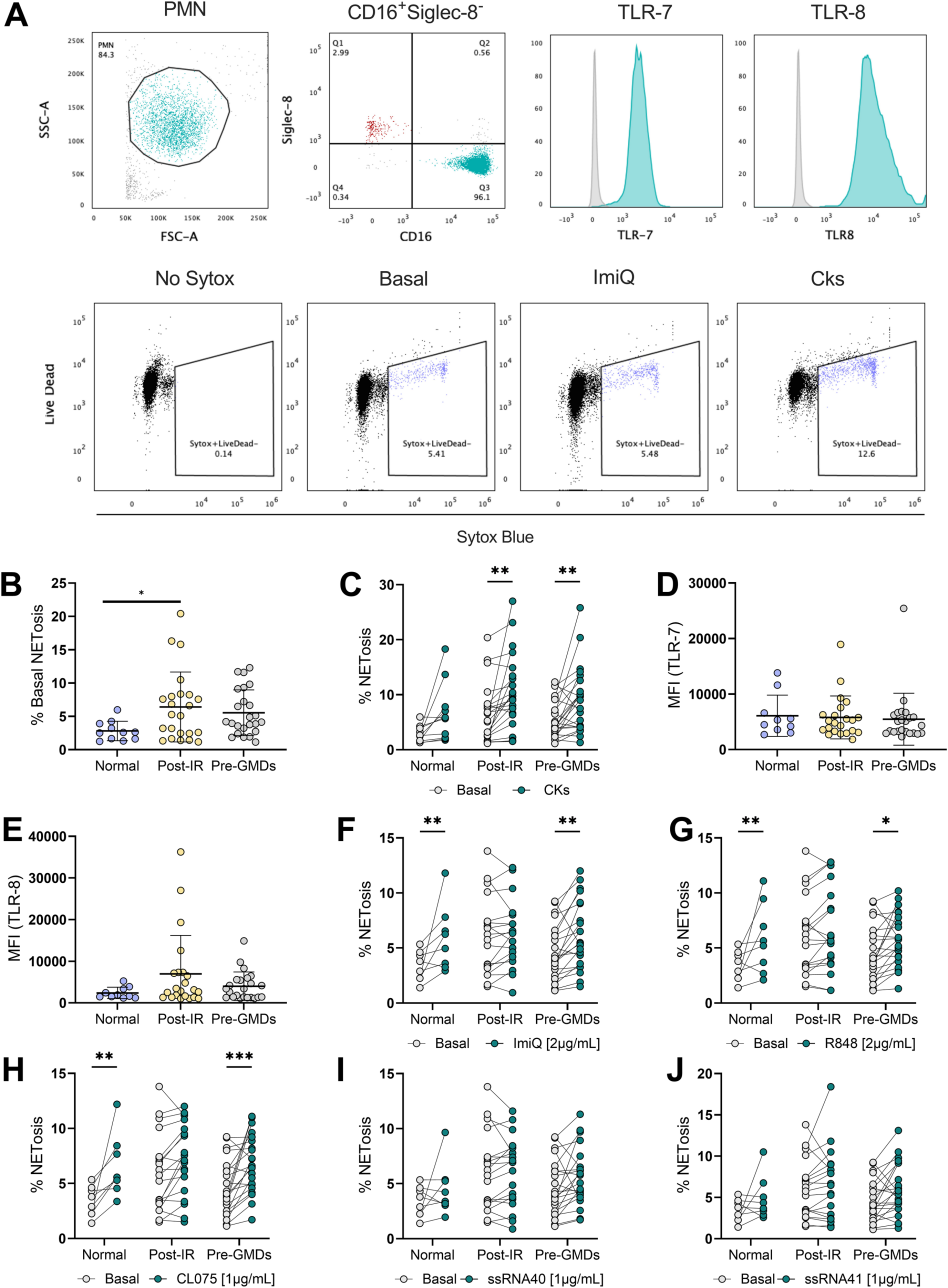
Evaluation of NETosis in neutrophils of the study cohort by flow cytometry. **(A)** Flow cytometry plot strategy for the identification of PMN populations, neutrophils (using CD-16/anti-Singlec8 staining), quantification of TLR7/8 and identification of NETosis (using Live/Dead Dye and Sytox Blue staining). **(B)** Scatter plots showing the quantification of the percentage of baseline NETosis in neutrophils from the three patient groups of the study cohort. Ordinary one-way ANOVA *p <0.05. **(C)** Paired data scatter plots comparing the percentage of baseline NETosis and when stimulated with IL-6 (20ng/mL) and TNFα (2ng/mL). 2-way ANOVA **p <0.01. Scatter plots showing the quantification of the expression of **(D)** TLR7 and **(E)** TLR8 according to median fluorescence intensity (MFI). Ordinary one-way ANOVA. **(F)** Scatter plots of paired data comparing the percentage of baseline NETosis against stimuli on TLR receptors such as ImiQ (2μg/mL), **(G)** R848 (2μg/mL), **(H)** CL075 (1μg/mL), **(I)** ssRNA40 (1μg/mL) and **(J)** ssRNA41 (1μg/mL). For panels **(F–J)**, 2-way ANOVA *p <0.05, **p <0.01 ***p <0.005.

Baseline and post-stimulation NETosis were evaluated in fresh isolated neutrophils from patients at 4 months post-COVID-19. Within the PMN population, neutrophils were identified as CD16^+^Singlec8^-^, TLR7/8 expression was measured from the PMN population and vital NETosis was identified as Live/Dead^-^SytoxBlue^+^ cells (**Figure 2A**). We observed a significant increment in basal NETosis in neutrophils from patients with IR post-COVID-19 compared to the other groups (**Figure 2B**). Then, NETosis was evaluated after IL-6 and TNF-α stimulation. An increase in NETosis was observed in the groups with pre-GMDs or post-viral IR compared to patients with no alteration (**Figure 2C**). We then measured TLR7 and TLR8 intracellular expression in isolated neutrophils from patients. No significant differences for TLR7 (**Figure 2D**) or TLR8 (**Figure 2E**) intracellular expression between groups was observed. Then, NETosis was evaluated after stimulation with TLR7/8 agonist to mimic viral ssRNA recognition. When, NETosis was evaluated in response to imiquimod (ImiQ), a TLR7 agonist, a significant increase in the percentage of NETosis was observed in neutrophils from patients without GMDs, but not in patients who develop IR post-COVID-19, when compared to baseline NETosis (**Figure 2F**). Similar results were observed when Resiquimod (R848) (**Figure 2G**) and CL075 (**Figure 2H**), simultaneous agonists of TLR7 and TLR8 were used. TLR8 stimulation with ssRNA40 (**Figure 2I**) and ssRNA41 (**Figure 2J**), did not result in differences in post-stimulation NETosis compared to baseline NETosis. These data suggest that patients who develop IR post-COVID-19 have dysregulated NETosis in comparison with the control group, exacerbating NETosis at basal levels and in response to cytokines, but not with viral-related stimuli. Patients with GMDs prior COVID-19 are difficult to interpret as these patients regulate their metabolic parameters with pharmacological drugs and have different types of GMDs, whereas *de novo* IR did not use medication prior to our evaluation.

### 3.3 Plasma from patients with IR produces an increase in NETosis in healthy neutrophils

**Figure 3:**
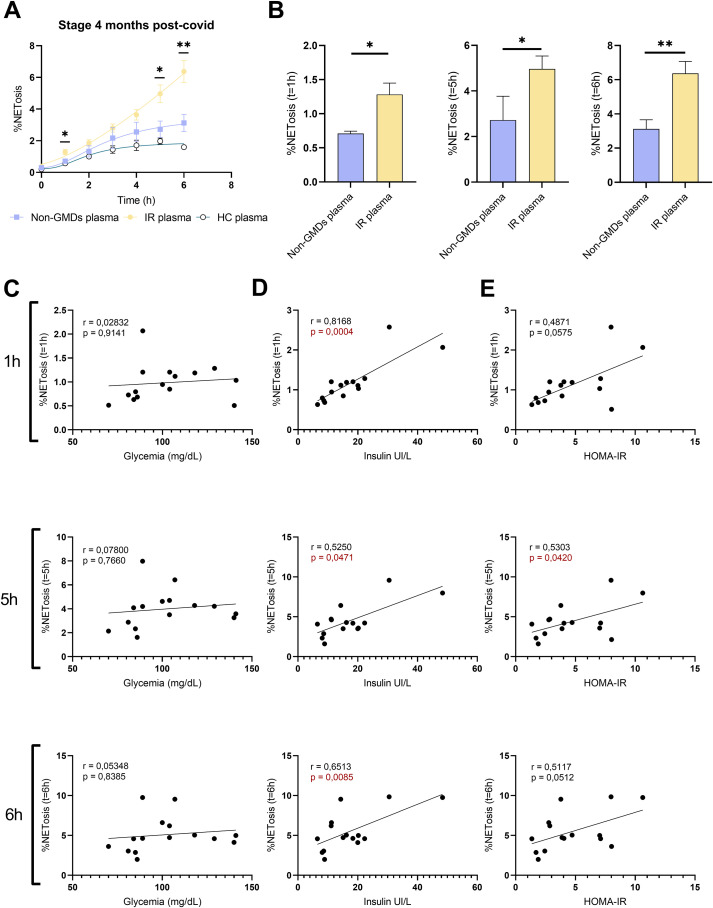
NETosis assessment in response to plasma from patients using IncuCyte. **(A)** Representative sigmoidal curve from IncuCyte based on the percentage of NETosis in healthy neutrophils exposed for 0 to 6 hours to plasma from normal patients 4 months post-COVID-19 (non-GMDs plasma 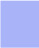), plasma from patients with IR 4 months post-COVID-19 (IR-plasma 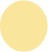) and plasma from healthy controls (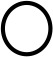). **(B)** Representative bar graphs comparing the percentage of NETosis in healthy neutrophils exposed to plasma for 1, 5 and 6 hours from normal patients (n=4) and plasma from patients with IR (n=14). Mann-Whitney test *p <0.05, **p <0.01. **(C)** Correlation graph representing the linearity of the data corresponding to the percentage of NETosis obtained at IncuCyte at 1, 5 and 6 hours using non-IR plasma and IR plasma with respect to the data obtained at 4 months post-COVID-19 for Glycemia, **(D)** Insulinemia and **(E)** HOMA-IR. significance *p <0.05.

Since neutrophils from the *de novo* IR group responded to cytokine stimulation, we excluded an intrinsic cellular defect in NETosis induction in these patients, therefore we assessed whether plasma factors contributed to NETosis. NETosis quantification based on a kinetic curve from 0 to 6 hours allowed us to evaluate the induction of NETosis by plasma from patients with IR or without GMDs (**Figure 3A**). An increment in the percentages of NETosis of healthy neutrophils after 1, 5 and 6 hours of exposure with plasma from patients with IR in comparison with patients without IR was observed (**Figure 3B**). The effect of plasma obtained during the acute phase of these patients on healthy neutrophils was also evaluated, however no significant differences were found, despite the higher levels of cytokines in these plasmas (**Supplementary Figure 1**), suggesting that NETosis was associated with IR rather than the presence high levels of pro inflammatory cytokines. Finally, NETosis was correlated with clinical parameters obtained 4 months post-COVID-19 in the group of patients with IR. No correlation between the percentage of NETosis and blood glucose was observed (**Figure 3C**). In contrast, the correlations between NETosis and insulin (**Figure 3D**) and NETosis and HOMA-IR index (**Figure 3E**) showed positive linearity and remained significant after 1, 5 and 6 hours of exposure.

### 3.4 Insulin plays an enhancing role in NETosis

**Figure 4:**
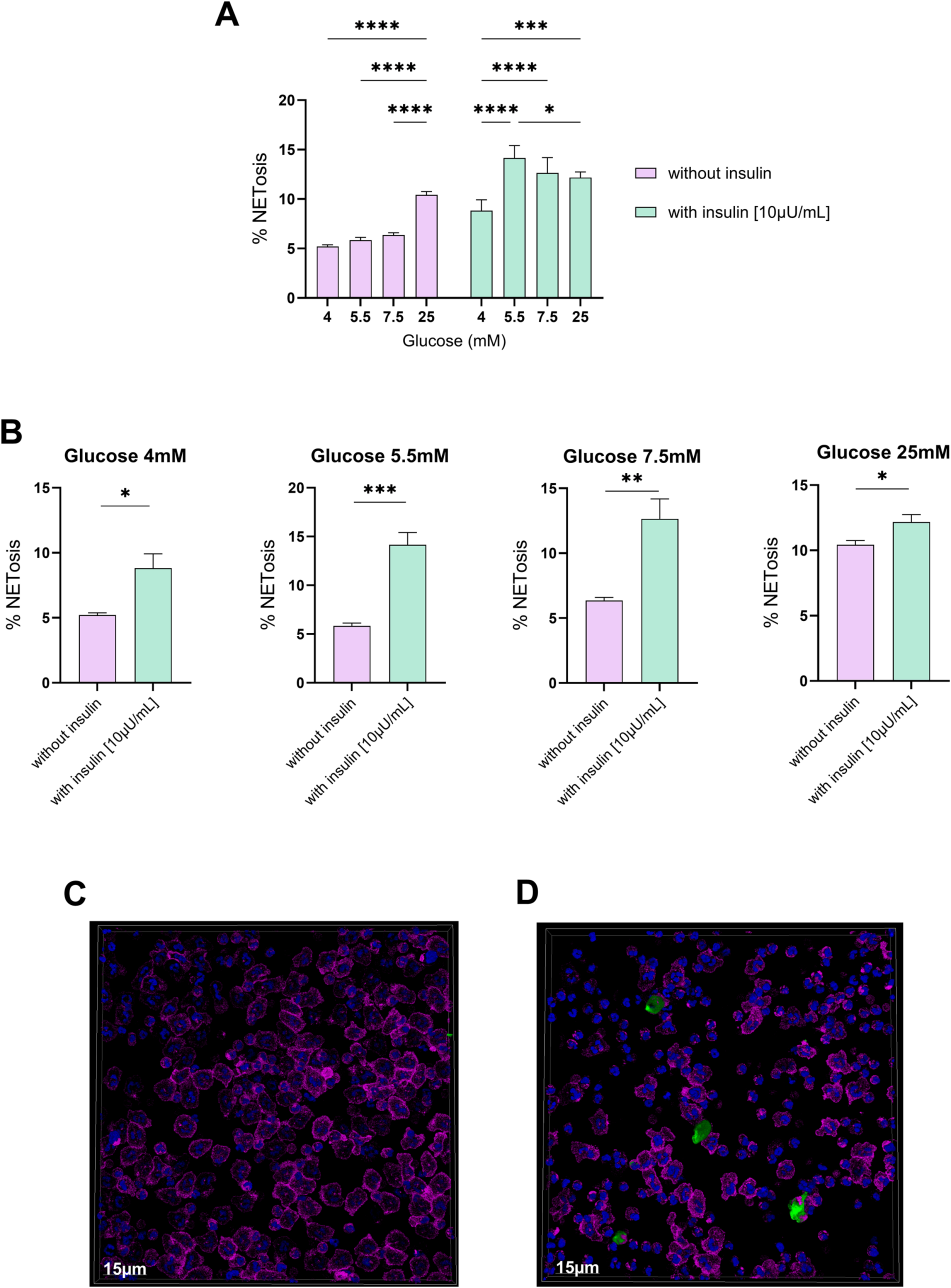
Evaluation of NETosis using glucose in the absence or presence of insulin. **(A)** Bar chart representing the different glucose concentrations (4 mM, 5.5 mM, 7.7 mM, and 25 mM) used in the culture of healthy neutrophils (n=6) in the absence and presence of insulin (10 µU/mL), evaluating the percentage of NETosis in each case. 2-way ANOVA *p <0.05, **p <0.01 ***p <0.005, *p <0.0001. **(B)** Bar charts showing the direct difference in the percentage of NETosis between the culture of healthy neutrophils (n=6) with different glucose concentrations in the absence or presence of insulin. Paired t-test *p <0.05, **p <0.01 ***p <0.005. **(C)** Comparison of NETosis by confocal microscopy with healthy neutrophils cultured with glucose (5.5 mM) in the absence of insulin or **(D)** in the presence of insulin (10 µU/mL). Measurements were performed between 10 and 60 minutes after culture conditions. Hoechst was used as a nuclear stain (blue), Sytox Green was used as a marker for NETosis, and CellMask was used to label the plasma membrane. The images correspond to real-time live-cell confocal microscopy acquired with a 63X 1.4 NA objective. ****p < 0.0001.

Finally, to test the influence of glucose and insulin on the environment associated with NETosis production, healthy neutrophils were used as biosensors against four different glucose concentrations that mimic plasma glucose conditions in the presence or absence of insulin. Once the cells were cultured under the different conditions, the percentages of NETosis were quantified after 10 to 60 minutes (**Figure 4A**). This revealed a significant increase in NETosis in neutrophils exposed to glucose and insulin compared to those exposed to glucose in the absence of insulin (**Figure 4B**). While the absence of insulin and the presence of glucose alone cause an increase in NETosis, this only occurs at very high glucose concentrations (25 mM) (**Figure 4A**), which is neither physiologically possible nor observed within the characteristics of our cohort. These data suggest that the presence of insulin enhances the NETosis process, which resembles the systemic environment observed in patients with insulin resistance in our cohort, who maintain normal glucose levels at the expense of elevated blood insulin levels. Finally, to confirm the influence of insulin on NETosis, a visual comparison was performed using confocal microscopy with healthy neutrophils in the presence of glucose (4mmol/L) without insulin (**Figure 4C**) and with insulin (10µU/mL) (**Figure 4D**). This comparison revealed a marked increase in NETosis, as seen with Sytox Green staining, in the sample containing insulin compared to the sample of neutrophils treated only with glucose.

## 4 Discussion

This study characterized the emergence of GMDs following COVID-19 and assessed their impact on NETosis. First, we observed *de novo* IR following SARS-CoV-2 infection in 24 patients without pre-existing GMDs. It has been widely described that COVID-19 survivors can experience metabolic or physical alterations beyond 12 weeks after SARS-CoV-2 infection, a condition known as long COVID-19 or post-COVID-19 syndrome (23, 24). Based on the post-COVID-19 syndrome in our cohort, it was possible to identify that the group that developed IR over time presented normal blood glucose levels, possibly due to significantly high levels of insulin in the blood. These findings are consistent with international reports that identify hyperglycemia and IR as frequent manifestations during COVID-19 (5, 6), although according to our data, the development of this GMDs problem is not related to ARDS. Furthermore, the HOMA-IR classification allowed this group of patients to be identified as insulin resistant (25), increasing their risk of metabolic decompensation. Regarding the biochemical profile, patients who developed IR presented elevated levels of AST, ALT, and AP, which could reflect damage to metabolically relevant organs such as the pancreas or liver tissues that have been identified as potential targets of SARS-CoV-2 infection (26, 27). Given that the liver and pancreas are important organs in the regulation of glycemia and insulinemia (28), these findings suggest a potential link between the infection and IR development (29). Our findings also highlight the dynamic nature of glucose metabolism alterations following COVID-19 between 4 and 12 months. While a reduction in the number of patients with insulin resistance (IR) was observed at 12 months, a subset of individuals developed *de novo* IR during follow-up, underscoring the heterogeneity of metabolic trajectories within this cohort. Notably, patients were advised at the 4-month evaluation to seek medical follow-up; therefore, the improvement observed in some individuals may be partly explained by the initiation of pharmacological treatment and/or lifestyle modifications. Conversely, variability in adherence to these recommendations may have contributed to the persistence or emergence of IR in others. In addition, post-pandemic factors, including changes in physical activity, dietary habits, and psychosocial stress, may have further influenced metabolic outcomes. Although causality cannot be established, these findings emphasize the need for longitudinal monitoring of glucose metabolism in post-COVID-19 patients and support the notion that these alterations may be, at least partially, reversible.

Stimuli known to induce NETosis include microbial pathogens, pathogen-associated molecular patterns (PAMPs) such as lipopolysaccharide (LPS), inflammatory cytokines (e.g., IL-6, IL-8), immune complexes, activated platelets, and glucose. Both insulin and cytokines were increased in plasma from patients with *de novo* IR. The cytokine storm has been described as part of the exacerbated immune response during the acute phase of COVID-19 (30). IL-6 is the main regulator of inflammatory proteins in the liver (31). When cytokines were analyzed in our cohort, a significant increase in IL-6 was observed in patients who developed post-COVID-19 IR, reinforcing the association between this cytokine and systemic inflammation in COVID-19 (32). Similarly, the increase in IL-8 in patients who developed IR compared to those who did not align with studies linking it to a worse prognosis in the acute phase of the disease (33), and with its potential role as a biomarker of inflammatory states associated with IR (34). These cytokines have been associated with inflammatory states linked to obesity and IR (34, 35). Interestingly, despite having higher IL-6/8 levels in the IR group at 4 months post-COVID-19 than control patients in circulation, we did not observe higher percentages of NETosis when plasma from patients during their acute phase were used, despite having substantially higher levels of IL-6/8 compared to 4 months post-COVID-19 (**Supplementary Figure 2**). These findings indicate that elevated IL-6/8 levels are unlikely to directly drive increased basal NETosis in the IR group, in contrast to the effect observed for insulin.

Our findings reveal a functional uncoupling in neutrophils from post-COVID-19 patients who develop insulin resistance, characterized by enhanced NETosis and increased responsiveness to cytokine stimulation, alongside a diminished response to TLR7 agonists. Accumulating evidence indicates that neutrophils from patients with COVID-19 display a pre-activated phenotype with increased propensity to form neutrophil extracellular traps (NETs), largely driven by elevated basal reactive oxygen species (ROS) and PAD4-dependent chromatin decondensation (12, 36). This primed state effectively lowers the activation threshold required for NETosis, thereby amplifying responses to cytokines such as TNF-α and IL-1β, which signal through NF-κB and MAPK pathways independently of endosomal pattern recognition (37, 38). In contrast, the reduced responsiveness to TLR7 stimulation is consistent with the establishment of innate immune tolerance following sustained viral sensing. TLR tolerance is characterized by downregulation of receptor expression and proximal signaling components such as MyD88 and IRAK4, together with upregulation of negative regulators including A20 and IRAK-M, ultimately limiting downstream type I interferon responses upon restimulation (39). Importantly, metabolic alterations associated with insulin resistance may further reinforce this phenotype by sustaining chronic low-grade inflammation and ROS production, thereby maintaining neutrophil priming while selectively impairing pathogen-sensing pathways (40). Collectively, these data support a model in which post-COVID neutrophils undergo maladaptive innate immune reprogramming, characterized by a reduced activation threshold for effector responses such as NETosis and cytokine-driven activation, coupled to a selective impairment in TLR7-dependent antiviral signaling.

Regarding NETosis induction, an increased basal NETosis in patients who developed IR was observed. Since we observed that plasma from post-COVID-19 IR patients induced increased NETosis by healthy neutrophils, we proposed that insulin could potentiate the NETosis process. This is clinically relevant as NETosis favors thrombus formation because NETs inhibit natural anticoagulants, in addition to promoting kidney damage and autoimmune diseases (41–43). Therefore, these changes could explain the greater predisposition to thrombosis in COVID-19 (44–47) patients with diabetes or prediabetes and their worse prognosis. While inflammatory components have been proposed as key players in the regulation of NETosis (48), we demonstrate that insulin plays a predominant role and may be crucial in stimulating the molecular pathways associated with NET formation.

This modulation of neutrophils by insulin could be effective directly or indirectly, considering that our study is still preliminary and that the underlying mechanisms still need to be thoroughly investigated. Based on this, it is possible that insulin acts on NETosis through multiple pathways. Several studies have demonstrated that elevated glucose concentrations promote the release of NETosis, positioning hyperglycemia as a key stimulus for neutrophil activation (49, 50). However, our results suggest that glucose alone is not the main inducer of NETosis in this context. It has been reported that neutrophil metabolism is not based on ATP production through oxidative phosphorylation, and they obtain energy mainly through glycolysis (51). It has been described that glucose transport in PMNs can vary depending on the metabolic state. At rest, GLUT1 dominance is observed, with no response to insulin, but this changes in an activated pro-inflammatory state, where they become insulin sensitive by translocating GLUT-4 to their cell membrane (52). The signaling pathways linked to the insulin receptor (InR) in neutrophils are widely associated with neutrophil metabolic activation, regulating chemotaxis through PI3-K (53). The InR is also related to the MAPK pathway, which is associated with neutrophil adhesion (54). Furthermore, the involvement of the IGF-1 receptor cannot be overlooked, as it shares structural and functional similarities with the InR (55). These similarities may result in overlapping signaling effects, activating partially shared signaling pathways, even though the literature states that they have a unique biological effect (56, 57), meaning we cannot attribute the effect of insulin to a single receptor. It is also important to highlight that insulin is a key regulator of glucose metabolism, so it could modulate glucose uptake by neutrophils or even generate changes in neutrophil metabolic states that directly affect NET formation (49, 58). In addition, insulin has been described as influencing neutrophil phagocytic and bactericidal capacity (59).

Taken together, these findings suggest that insulin is emerging as a potential modulator of neutrophil metabolism and function, exerting a potentiating effect on the formation of NETs. This finding is reinforced by results obtained using confocal microscopy and live imaging, where plasma from patients with IR induced greater NETosis compared to plasma from control patients. Taken together, these data suggest that insulin may play a key role in neutrophil activation, possibly even surpassing the effect of cytokine-mediated inflammatory stimuli in the context of COVID-19 associated with metabolic dysfunction. Based on these conclusions, it becomes crucial to continue evaluating neutrophil metabolic pathways related to NETosis in the presence of IR. Furthermore, patients with COVID-19 who develop IR should be closely monitored, especially regarding thrombotic events.

Among the limitations of this study, it is important to note that dividing the cohort into different subgroups introduces some bias, limiting correlations, as the group identified with *de novo* insulin resistance comprises only 24 of the 60 patients in the study. Therefore, this study should be considered exploratory, suggesting that our conclusions require validation in larger, independent cohorts. Another limitation of our study is the relatively restricted characterization of the metabolic phenotype. Insulin resistance was primarily assessed using the HOMA-IR index, and although this proxy marker is widely used and validated in epidemiological and clinical research, it does not provide the same level of accuracy as reference methods, such as the hyperinsulinemic-euglycemic clamp test. Furthermore, oral glucose tolerance tests (OGTTs), which allow for a more comprehensive assessment of glucose handling and dynamic insulin responses, were not performed. Consequently, our assessment does not fully encompass the complexity of insulin sensitivity and glucose metabolism throughout the body. Despite these limitations, the HOMA-IR index has been shown to correlate reasonably well with measurements obtained using the clamping technique in various populations and remains a practical and accepted method for estimating insulin resistance in studies of this nature (60, 61). Furthermore, we have made available the data corresponding to serum insulin levels (**Supplementary Table 4**) and waist circumference (**Supplementary Table 5**) to support our selection of participants for the group without GMDs and the group with post-COVID-19 insulin resistance (25). Future studies incorporating direct and dynamic metabolic assessments will be important to further validate and expand upon our findings.

## Data Availability

The original contributions presented in the study are included in the article/**Supplementary Material**. Further inquiries can be directed to the corresponding author.
