# Supplementary material for: De novo COVID-19-associated insulin resistance drives dysregulated neutrophil extracellular trap formation (NETosis) four months after infection
Source: Front Immunol. 2026 May 4;17:1787799. doi: 10.3389/fimmu.2026.1787799 (PMC13182236; doi:10.3389/fimmu.2026.1787799)
Supplement: Supplementary file 1 [file Supplementaryfile1.pdf]

Supplementary Material

De novo COVID-19-Associated Insulin Resistance Drives Dysregulated Neutrophil Extracellular Trap Formation (NETosis) Four Months After Infection

Running title: Post-COVID *de novo* IR affects NETosis

Sergio Sanhueza[1 †], Camilo D. Cabrera[1 †], Romina A. Quiroga[1], Bárbara E. Antilef[1], Camila P. Muñoz[1,2], Agustín A. Vera[1], Ricardo Cartes[1,3], Liliana I. Lamperti[1], Enrique Guzmán-Gutiérrez[1], Claudio Aguayo[1], Valeska Ormazábal[1], Mauricio A. Hernández[4], Jaime J. Lastra[5], Benilde Riffo[6], Gustavo Cerda[7], Luciano Ferrada[7], David de Gonzalo-Calvo[8,9], María C. García-Hidalgo [8,9], Mario Henríquez-Beltrán[10,11,12], María Inés Barría[13], Ricardo A. Verdugo[14], Alicia Colombo[15], Gonzalo Labarca[1,12,16,17] & Estefanía Nova-Lamperti[1]*

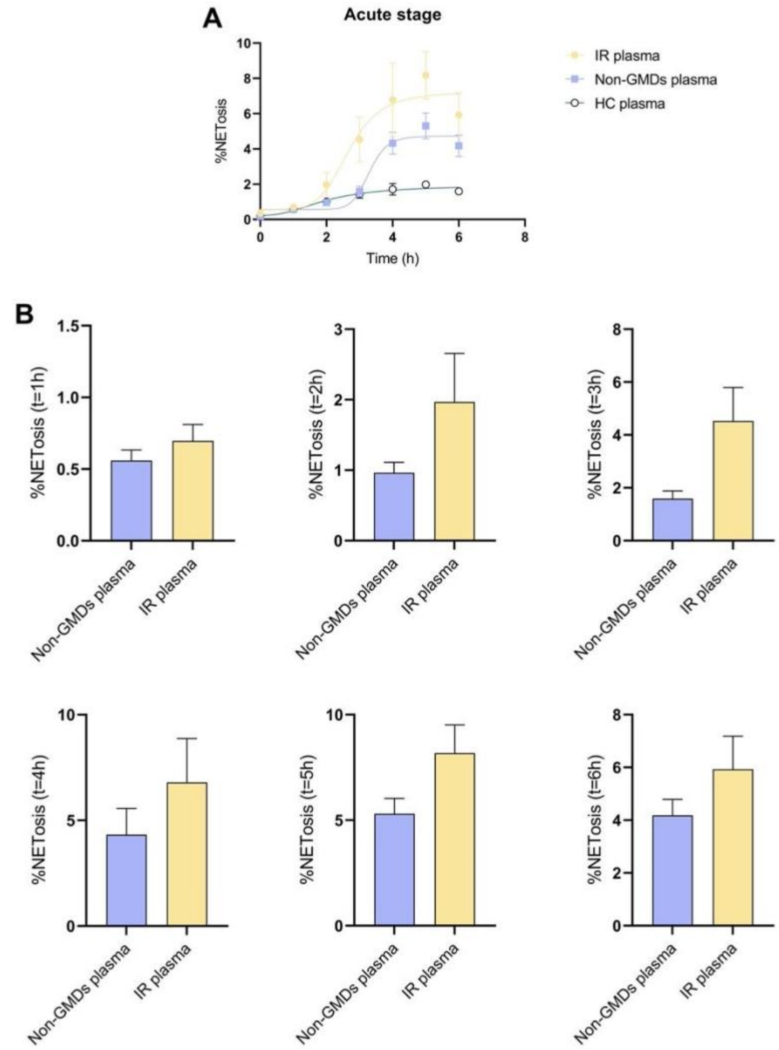

Supplementary Figure 1. NETosis assessment using IncuCyte at acute state of patients with COVID-19. (A) Representative sigmoid curve of Incucyte based on the percentage of NETosis in healthy neutrophils exposed for 0 to 6 hours to normal patient plasma (non-GMDs) in acute stage (■), plasma from patients with IR in acute stage (●) and plasma from healthy controls (○). **(B)** Representative bar graphs comparing the percentage of NETosis in healthy neutrophils exposed to plasma for 1, 5 and 6 hours from normal patients (n=4) and plasma from patients with IR (n=14). Mann-Whitney test.

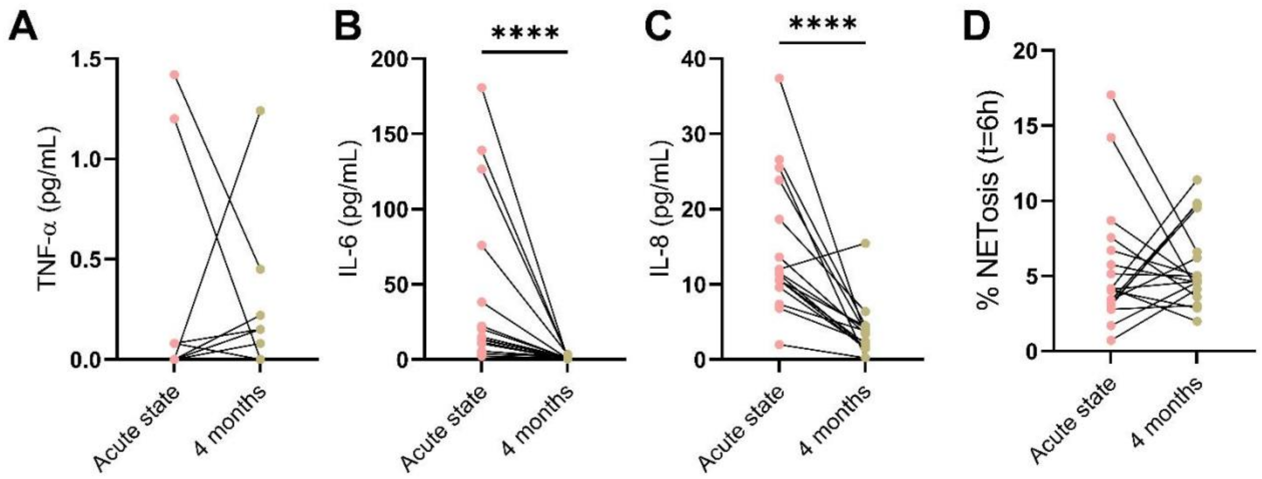

Supplementary Figure 2. Comparison of cytokines and NETosis quantified from the acute state and 4 months post COVID-19. Comparison of the quantification of cytokines present in plasma obtained during the acute stage and 4 months post-COVID-19 (n=16) **(A)** TNF-α, **(B)** IL-6 and **(C)** IL-8. **(D)** Comparison of the percentage of Netosis obtained by IncuCyte after 6 hours of culture using plasma from patients without GMDs and with post-COVID-19 IR (n=18) collected during the acute stage and 4 months post-COVID-19. Wilcoxon signed-rank test ****p <0.0001.

Supplementary Table 1. Clinical characteristics of the study cohort.

	Patients Without GMDs	Patients with Insulin resistance post COVID-19	Patients with GMDs pre COVID-19	*p*-Value
**Gender**				
Male:female, *n* (%)	6:6 (50:50)	14:10 (58.3:41.7)	12:12 (50:50)	n.s
**ABO group**				n.s
A, *n* (%)	4 (33.3)	4 (16.7)	6 (25)	n.s
B, *n* (%)	1 (8.3)	2 (8.3)	3 (12.5)	n.s
AB, *n* (%)	0 (0)	1 (4.2)	1 (4.2)	n.s
O, *n* (%)	7 (58.4)	17 (70.8)	14 (58,3)	n.s
**Measurements**				
Weight, Kg (SD)	76.4 ± 14.6	83.4 ± 14.5	89.2 ± 15.4	*
Height, m (SD)	1.66 ± 0.1	1.66 ± 0.1	1.62 ± 0.1	n.s
BMI, Kg/m^2	27.6 ± 4.7	29.9 ± 3.8	34.0 ± 4.6	***

Neck circumference, cm (SD)	39 ± 4.7	41.3 ± 5.0	43.9 ± 5.1	*
Waist circumference, cm (SD)	95.5 ± 11.7	99.9 ± 10.8	111.5 ± 11.1	***
Hip circumference, cm (SD)	101.4 ± 7.5	108.7 ± 8.8	112 ± 9.3	**
**Tobacco status**				n.s
Current, *n* (%)	3 (25)	3 (12.5)	2 (8.3)	n.s
Former, *n* (%)	1 (8.3)	5 (20.8)	9 (37.5)	n.s
Never smoker, *n* (%)	8 (66.7)	16 (66.7)	13 (54.2)	n.s
**Alcohol usage**				n.s
Never, *n* (%)	3 (25)	10 (41.7)	12 (50)	n.s
Occasionally, *n* (%)	8 (66.7)	13 (54.2)	12 (50)	n.s
Frequently, *n* (%)	1 (8.3)	1 (4.1)	0 (0)	n.s
**COVID-19 severity**				n.s
Mild, *n* (%)	6 (50)	9 (37.5)	3 (12.5)	*
Moderate, *n* (%)	1 (8.3)	5 (20.8)	11 (45.8)	*
Severe/critical, *n* (%)	5 (41.7)	10 (41.7)	10 (41.7)	n.s

ARDS, *n* (%)	4 (33.3)	12 (50)	18 (75)	*
**Symptoms during acute phase**				
Fever, *n* (%)	6 (50)	15 (62.5)	15 (62.5)	n.s
Headache, *n* (%)	6 (50)	17 (70.8)	14 (58.3)	n.s
Chest pain, *n* (%)	7 (58.4)	9 (37.5)	11 (45.8)	n.s
Sore throat, *n* (%)	5 (41.7)	9 (37.5)	12 (50)	n.s
Cough, *n* (%)	6 (60)	15 (62.5)	18 (75)	n.s
Dyspnea, *n* (%)	7 (58.4)	17 (70.8)	20 (83.3)	n.s
Polypnea, *n* (%)	4 (33.3)	15 (62.5)	17 (70.8)	n.s
Myalgia, *n* (%)	10 (83.3)	15 (62.5)	14 (58.4)	n.s
Desaturation, *n* (%)	1 (8.3)	1 (4.1)	1 (4.2)	n.s
Abdominal pain, *n* (%)	4 (33.3)	8 (33.3)	7 (29.2)	n.s
Diarrhea, *n* (%)	4 (33.3)	10 (41.7)	7 (29.2)	n.s
Change smell, *n* (%)	5 (41.7)	10 (41.7)	10 (41.7)	n.s
Change taste, *n* (%)	6 (50)	10 (41.7)	8 (33.3)	n.s

**Comorbidities**				
Arterial hypertension, *n* (%)	3 (25)	3 (12.5)	14 (58.3)	**
IR at baseline, *n* (%)	0 (0)	0 (0)	12 (50)	****
T2DM at baseline, *n* (%)	0 (0)	0 (0)	8 (33.3)	***
Heart failure, *n* (%)	0 (0)	0 (0)	0 (0)	n.s
COPD, *n* (%)	0 (0)	0 (0)	0 (0)	n.s
Previous cancer, *n* (%)	0 (0)	0 (0)	1 (4.2)	n.s
CKD, *n* (%)	0 (0)	0 (0)	0 (0)	n.s
Afib, *n* (%)	0 (0)	0 (0)	1 (4.2)	n.s
Stroke, *n* (%)	0 (0)	0 (0)	1 (4.2)	n.s
CHD, *n* (%)	0 (0)	0 (0)	0 (0)	n.s
NAFLD, *n* (%)	0 (0)	5 (20.8)	2 (8.3)	n.s
Hypothyroidism, *n* (%)	0 (0)	1 (4.1)	4 (16.7)	n.s
Hepatic Steatosis, *n (%)*	0 (0)	6 (25)	11 (45.8)	*
**Therapy**				
ACE/ARBs, *n* (%)	1 (8.3)	2 (8.3)	9 (37.5)	*

Beta blockers, *n* (%)	0 (0)	0 (0)	3 (12.5)	n.s
Ca^{++} blq, *n* (%)	1 (8.3)	0 (0)	5 (20.8)	n.s
Potassium sparing diuretics, *n* (%)	0 (0)	0 (0)	1 (4.2)	n.s
Thiazide drugs, *n* (%)	0 (0)	0 (0)	4 (16.7)	*
Metformin, *n* (%)	0 (0)	0 (0)	16 (66.7)	****
Insulin, *n* (%)	0 (0)	0 (0)	6 (25)	**
Hyperlipidemia drug, *n* (%)	0 (0)	0 (0)	12 (50)	****
Z drugs	0 (0)	4 (16.7)	6 (25)	n.s
IRSS	0 (0)	2 (8.3)	3 (12.5)	n.s
**4-months after COVID-19**				
Pulmonary test				
Abnormal CT, *n* (%)	5 (41.7)	11 (45.8)	21 (87.5)	**
DLCO <80%, *n* (%)	1 (8.3)	6 (25)	12 (50)	*
**Symptoms**				
Fever, *n* (%)	0 (0)	0 (0)	0 (0)	n.s

Headache, *n* (%)	5 (41.7)	9 (37.5)	7 (29.2)	n.s
Chest pain, *n* (%)	2 (16.7)	1 (4.2)	1 (4.2)	n.s
Sore throat, *n* (%)	1 (8.3)	2 (8.3)	2 (8.3)	n.s
Cough, *n* (%)	1 (8.3)	4 (16.7)	7 (29.2)	n.s
Dyspnea, *n* (%)	2 (16.7)	4 (16.7)	9 (37.5)	n.s
Polypnea, *n* (%)	0 (0)	1 (4.2)	3 (12.5)	n.s
Myalgia, *n* (%)	1 (8.3)	3 (12.5)	3 (12.5)	n.s
Abdominal pain, *n* (%)	0 (0)	1 (4.2)	0 (0)	n.s
Change smell, *n* (%)	1 (8.3)	2 (8.3)	1 (4.2)	n.s
Change taste, *n* (%)	0 (0)	1 (4.2)	0 (0)	n.s

a) BMI, body mass index; b) ARDS, acute respiratory distress syndrome; c) IR, insulin resistance; d) T2DM, type 2 diabetes mellitus; e) COPD, chronic obstructive pulmonary disease; f) CKD, chronic kidney disease; g) Afib, atrial fibrillation; h) CHD, coronary heart disease; i) NAFLD, non-alcoholic fatty liver disease. j) ACE, angiotensin-converting enzyme; k)ARBs, Angiotensin II Receptor Blockers; l) *n*, number of patients; m) %, percentage; n) SD, standard deviation. Chi-square test; ****$p < 0.0001$, ***$p < 0.005$, **$p < 0.01$, *$p < 0.05$.

Supplementary Table 2. Cohort evolution according to HOMA-IR.

Patient ID	Group	Age	Gender	HOMA-IR 4m	HOMA-IR 12m
COVID1005-3	Without GMDs	45	1	1.9	3.6
COVID1005-4	Without GMDs	41	0	2.0	1.5
COVID1005-6	Without GMDs	23	0	2.2	3.5
COVID1005-7	Without GMDs	36	0	2.1	4
COVID1005-13	Without GMDs	24	1	2.5	Missing
COVID1005-28	Without GMDs	74	1	1.3	5.8
COVID1005-32	Without GMDs	25	0	1.8	Missing
COVID1005-34	Without GMDs	54	0	1.7	2.2
COVID1005-46	Without GMDs	37	0	1.7	1.8
COVID1005-47	Without GMDs	38	1	2.5	3
COVID1005-49	Without GMDs	64	1	1.9	1.6
COVID1005-59	Without GMDs	45	1	2.0	2.5
COVID1005-1	IR post COVID-19	33	1	3.3	3.8
COVID1005-2	IR post COVID-19	64	0	2.7	1.2
COVID1005-8	IR post COVID-19	41	0	3.9	3.6
COVID1005-9	IR post COVID-19	30	1	3.6	2.6
COVID1005-10	IR post COVID-19	39	1	3.6	3.9
COVID1005-11	IR post COVID-19	34	0	2.6	3.2
COVID1005-12	IR post COVID-19	43	1	3.9	3.0
COVID1005-16	IR post COVID-19	55	1	2.8	2.2
COVID1005-18	IR post COVID-19	33	1	4.5	2.2
COVID1005-19	IR post COVID-19	60	1	4.1	4.1
COVID1005-21	IR post COVID-19	41	0	5.8	Missing
COVID1005-23	IR post COVID-19	33	1	2.6	1.1
COVID1005-24	IR post COVID-19	54	0	9.5	3.7
COVID1005-25	IR post COVID-19	33	0	2.9	2.1
COVID1005-30	IR post COVID-19	27	1	3.7	2.3
COVID1005-31	IR post COVID-19	40	0	6.0	2.4
COVID1005-35	IR post COVID-19	41	1	2.9	3.7
COVID1005-36	IR post COVID-19	49	1	2.8	3.4
COVID1005-37	IR post COVID-19	53	1	3.8	3.5
COVID1005-38	IR post COVID-19	27	0	5.6	Missing
COVID1005-40	IR post COVID-19	20	1	6.0	5.2
COVID1005-44	IR post COVID-19	27	1	4.0	5.8
COVID1005-45	IR post COVID-19	60	0	5.0	5.7
COVID1005-60	IR post COVID-19	48	0	5.1	2

Supplementary Table 3. Circulatory parameters.

	**Patients Without GMDs**	**Patients with Insulin resistance post COVID-19**	***p*-Value**
**Other parameters (U/L)**			
Uric acid (SD)	4.792 (±1.087)	5,188 (±1,488)	n.s
Calcium (SD)	7.433 (±2.685)	8.579 (±1.765)	n.s
Phosphorus (SD)	3.353 (±0.4769)	3.685 (±0.6513)	n.s
Total bilirubin (SD)	0.5754 (±0.1791)	0.5252 (±0.2127)	n.s
Direct bilirubin (SD)	0.2615 (±0.1371)	0.2279 (±0.1049)	n.s
Indirect bilirubin (SD)	0.3138 (±0.1422)	0.3000 (±0.1485)	n.s
Total protein (SD)	6.423 (±1.320)	6.988 (±0.9502)	n.s
Albumin (SD)	3.825 (±0.9037)	4.200 (±0.6440)	n.s
Globulins (SD)	2.525 (±0.5545)	2.788 (±0.5416)	n.s
**Blood count**			

Hemoglobin g/dL (SD)	14.77 (±1.657)	15.01 (±1.125)	n.s
Hematocrit % (SD)	42.23 (±4.342)	42.34 (±2.902)	n.s
Erythrocyte x10^6/L (SD)	4.720 (±0.5132)	4.677 (±0.4161)	n.s
MCV fL (SD)	89.63 (±3.966)	90.75 (±3.735)	n.s
MCH pg (SD)	31.36 (±1.739)	32.18 (±1.350)	n.s
MCHC g/dL (SD)	34.94 (±0.7763)	35.48 (±0.8546)	n.s
Leukocytes x10^6/L (SD)	5425 (±1498)	5717 (±1383)	n.s
Lymphocytes % (SD)	29.13 (±9.330)	29.01 (±7.101)	n.s
Monocytes % (SD)	5.527 (±0.8821)	6.525 (±1.903)	n.s
Granulocytes (SD)	64.30 (±8.069)	64.15 (±7.396)	n.s
**Lipid profile (mg/dL)**			
Total colesterol (SD)	197.3 (±44.21)	198.2 (±52.45)	n.s
Triglycerides (SD)	116.0 (±55.78)	112.7 (±40.44)	n.s
HDL (SD)	53.00 (±22.13)	51.88 (±16.67)	n.s

LDL (SD)	117.6 (±46.37)	114.4 (±39.00)	n.s

a) MCV, Mean Corpuscular Volume; b) MCH, Mean Corpuscular Hemoglobin; c) MCHC, Mean Corpuscular Hemoglobin Concentration; d) HDL, High-density lipoprotein; e) LDL, Low-density lipoprotein; f) *n*, number of patients; g) %, percentage; h) SD, standard deviation. Mann Whitney test; $^{*}p < 0.05$.

Supplementary Table 4. Insulinemia obtained 4 months post COVID-19.

Insulin without GMDs group (µU/mL)		Insulin post IR group (µU/mL)	
COVID1005-3	6.33	COVID1005-1	12.43
COVID1005-4	7.07	COVID1005-2	7.25
COVID1005-6	8.95	COVID1005-8	19.63
COVID1005-7	9.01	COVID1005-9	19.75
COVID1005-13	9.9	COVID1005-10	15.4
COVID1005-28	6.5	COVID1005-11	11.1
COVID1005-32	7.9	COVID1005-12	15.1
COVID1005-34	8.6	COVID1005-16	11.2
COVID1005-46	8.1	COVID1005-18	19.4
COVID1005-47	12.1	COVID1005-19	15.2
COVID1005-49	8.9	COVID1005-21	18.2
COVID1005-59	7.4	COVID1005-23	7.4
		COVID1005-24	27.8
		COVID1005-25	15.2
		COVID1005-30	16.1
		COVID1005-31	22.9
		COVID1005-35	11.1
		COVID1005-36	12.5
		COVID1005-37	14.3
		COVID1005-38	26.8
		COVID1005-40	23.8
		COVID1005-44	18.37
		COVID1005-45	20.1
		COVID1005-60	16.9

Supplementary Table 5. Waist circumference obtained 4 months post COVID-19.

Waist circumference without GMDs group (cm)		Waist circumference post IR group (cm)	
COVID1005-3	100	COVID1005-1	110
COVID1005-4	90	COVID1005-2	92
COVID1005-6	110	COVID1005-8	99
COVID1005-7	98	COVID1005-9	105
COVID1005-13	98	COVID1005-10	96
COVID1005-28	82	COVID1005-11	81
COVID1005-32	66	COVID1005-12	97
COVID1005-34	100	COVID1005-16	92
COVID1005-46	96	COVID1005-18	109
COVID1005-47	98	COVID1005-19	110
COVID1005-49	103	COVID1005-21	109
COVID1005-59	105	COVID1005-23	88
		COVID1005-24	104
		COVID1005-25	90
		COVID1005-30	83
		COVID1005-31	94
		COVID1005-35	106
		COVID1005-36	106
		COVID1005-37	101
		COVID1005-38	102
		COVID1005-40	111
		COVID1005-44	123
		COVID1005-45	109
		COVID1005-60	80
